# Life Cycle Assessment on Agricultural Production: A Mini Review on Methodology, Application, and Challenges

**DOI:** 10.3390/ijerph19169817

**Published:** 2022-08-09

**Authors:** Jianling Fan, Cuiying Liu, Jianan Xie, Lu Han, Chuanhong Zhang, Dengwei Guo, Junzhao Niu, Hao Jin, Brian G. McConkey

**Affiliations:** 1 Jiangsu Key Laboratory of Atmospheric Environment Monitoring and Pollution Control, Jiangsu Collaborative Innovation Center of Atmospheric Environment and Equipment Technology, School of Environmental Science and Engineering, Nanjing University of Information Science and Technology, 219 Ningliu Road, Nanjing 210044, China; 2 Jiangsu Key Laboratory of Agricultural Meteorology, School of Applied Meteorology, Nanjing University of Information Science and Technology, 219 Ningliu Road, Nanjing 210044, China; 3 Reading Academy, Nanjing University of Information Science and Technology, 219 Ningliu Road, Nanjing 210044, China; 4 Ministry of Agriculture and Agri-Food, Ottawa, ON K1A 0C5, Canada

**Keywords:** agricultural system, LCA methodology, LCA application, agricultural sustainability

## Abstract

Agricultural Life Cycle Assessment (LCA) is an effective tool for the quantitative evaluation and analysis of agricultural materials production and operation activities in various stages of the agricultural system. Based on the concept of life cycle, it comprehensively summarizes the impact of agriculture on the environment, which is an effective tool to promote the sustainability and green development of agriculture. In recent years, agricultural LCA has been widely used in the agroecosystem for resource and environmental impacts analysis. However, some challenges still exist in agricultural LCA, i.e., the environmental impact assessment index system needs to be improved; its application in different production mode is limited; and combination research with other models needs more attention. This paper discusses the above-mentioned challenges and recommends research priorities for both scientific development and improvements in practical implementation. In summary, further research is needed to construct a regional heterogeneity database and develop innovated methodologies to develop more meaningful functional units for agricultural products to complement LCA by other models. These efforts will make agricultural LCA more robust and effective in environmental impacts assessment to support decision making from individual farm to regional or (inter)national for the sustainable future of agriculture.

## 1. Introduction

In 1969, Coca-Cola Company used the life cycle assessment (LCA) method for the first time to comprehensively and quantitatively evaluate the resource consumption and environmental burdens generated in the whole life cycle of beverage bottles from raw material extraction to final disposal. In the 1980s, the LCA method was further explored in the industrial sector in order to fully understand the energy consumption. In 1993, the Society for Environmental Toxicology and Chemistry (SETAC) first proposed the concept of LCA, i.e., “LCA is a process to evaluate the environmental burdens associated with a product, process, or activity by identifying and quantifying energy and materials used and wastes released to the environment; to assess the impact of those energy and material uses and releases to the environment; and to identify and evaluate opportunities to affect environmental improvements” [1]. The International Organization for Standardization (ISO) then issued a series of standards on LCA in 1997 (ISO14040), 1999 (ISO14041), and 2000 (ISO14042 and ISO 14043), and further defined the LCA method as “A technique for assessing the environmental aspects and potential impacts associated with a product” [2].

The agricultural sector, providing a large number of products and services, is vital to human beings. The contradiction caused by low land productivity and excessive population density has seriously restricted the development of agriculture since the 20th century. In order to alleviate this contradiction to feed an increasing population, large-scale and high-intensity farming with a high input of chemical fertilizers, pesticides, and agricultural film were adopted in the process of pursuing the increase in productivity. However, on the other hand, this has led to serious negative environmental effects, including damage to natural resources, decreased land productivity, accelerated spread of pests and diseases, and a reduction in biodiversity [3,4]. The production and use of chemical fertilizers and pesticides also poses serious threats to food security and climate change, including the irrational use of water resources, eutrophication of water bodies, and a reduction in species genetic diversity. Furthermore, agriculture, as an important source of greenhouse gases (GHGs), accounts for 24% of the total global GHGs emissions according to IPCC Fifth Assessment Report [5], indicating that agriculture plays an important role in climate change. However, research on agricultural LCA methodology and its practical application is limited compared to the industry sector. Agricultural LCA, as an effective way to comprehensively and quantitatively assess resource consumption and environmental burdens in the whole process of agricultural production or agricultural activities, is an important method to promote the green and low-carbon development of agriculture and to achieve sustainable agricultural development. Therefore, the purposes of this paper were to discuss the remaining challenges in agricultural LCA and to recommend research priorities for both scientific development and improvements in practical implementation.

## 2. Methods

A systematic search of the scientific literature was carried out to find studies evaluating the environmental impacts of agricultural production. The checked database was ISI Web of knowledge (http://apps.webofknowledge.com). The used keywords were “agricultural LCA”, “Life Cycle Assessment agriculture”, or “Life Cycle Assessment crop production”. The search results were examined by title and abstract and the following selection criteria were applied: (i) The paper must be published in a peer-reviewed journal, (ii) The study must be related to agricultural production, and (iii) Only studies performing an impact assessment with more than one LCA indicator were retained.

## 3. Overview of Agricultural LCA Methodology

Research on agricultural LCA began in the mid-1990s, when the first academic seminar on agricultural LCA was organized in 1993. In 1998, Japan started the LCA methodology research for sustainable agriculture by organizing relevant research institutes affiliated to the Ministry of Agriculture, Forestry and Fisheries, agricultural enterprises and universities, which laid the foundation for the rapid development of agricultural LCA. Generally, the basic methodological framework of LCA consists of the following four parts: goal definition and scoping (G&S), life cycle inventory analysis (LCI), environmental impact assessment (LCIA), and interpretation of results [6].

### 3.1. Goal Definition and Scoping

When carrying out agricultural LCA, the first step is to define the research objectives and the system boundary, which is fundamental to the following steps and determines the validity of the assessment results. The system boundary contains the specific process of the LCA study to assess the environmental impact of the whole process, normally from cradle to grave (gate). As a systematic and comprehensive analysis method, agricultural LCA includes two dimensions: one is the dimension of the whole life cycle process, and the other is the impact of various resources and environments related to agricultural activities. These two dimensions also constitute the boundary of the system.

Another important aspect in this step is the functional unit (FU), which should be selected carefully since it is a measure of the output function of the product system. The FU could be based on area (such as 1 km^2^ field), product (such as 1 kg yield) or both together. It is suitable to compare the environmental effects when FU is selected based on area, while it is better to compare the production efficiency when FU is selected based on yield. However, there is increasing demand to use alternative FU to better represent the function and performance of a product. For instance, some studies propose using the nutritional value of the food in the FU [7,8] or FUs involving the social dimensions of products (e.g., wine, beers or coffee) [9,10]. Therefore, appropriate FU should be selected according to the research purposes, since different selected FU will lead to different results of environmental effects.

### 3.2. Life Cycle Inventory (LCI) Analysis

Life cycle inventory analysis, also known as agricultural environmental load analysis, is a data-based quantification process for all resource consumption and waste emissions related to functional units within the defined system boundary by using quantitative survey methods [11]. It is a basic step to carry out the work of LCA and a time-consuming and labor-intensive step to obtain relevant data on environmental impact and conduct a comprehensive quantitative analysis of the impact of the agricultural production process on both the ecological environment and human health. However, compared with the industrial LCA, the agricultural LCA is more complex in inventory analysis. This is reflected in the fact that the inventory data obtained by agricultural LCA are greatly affected by temporal and spatial conditions, which requires the collected data to be representative, consistent and comparable. Some real-scenario inventory data can be collected through field surveys, while relevant background data can only be obtained from the database. The reliability of the data is the premise for agricultural LCA work, where widely used databases include Ecoinvent (Switzerland) [12], ELCD (Europe) [13], GaBi (Germany) [14], NREL-USLCI (USA) [15], LCA Food (Denmark) [16], CLCD (China) [17], etc. Furthermore, the calculation of the potential environmental impacts in the LCI stage can be referred to the global, regional or site scale. They define the characterization factors that weigh all the substances contributing to a certain impact category and refer them to the indicator.

In summary, the following contents should be clarified in the inventory analysis stage: covering basic data (such as experimental data or simulated data), the assumptions made for agricultural production, the emission inventories used in model calculation, and emission factors and characterization factors of specific sites utilized.

### 3.3. Environmental Impact Assessment

The environmental impact assessment is based on the LCI results, to identify the impact of input-output of agricultural system on natural resources, human health and agricultural ecosystem health, which is the most controversial stage with the highest degree of difficulty among the four steps. In agricultural LCA, the impact of agricultural production on land resources should be taken into serious consideration since long-term agricultural practices will have a significant influence on soil quality. More attention should be paid to the following two aspects in the impact assessment stage: (1) determining which environmental impact categories to assess; and (2) determining which life-cycle impact assessment methods to use.

### 3.4. Interpretation

Results interpretation aims to conclude, summarize, and offer the proposal on the results of inventory analysis and impact assessment, and put forward corresponding suggestions for improvement of limitations [18]. The major steps include the identification, evaluation and report. Identification recognizes the problems throughout the course of the research based on the target of evaluation elements, the range of identification and special elements. Evaluation is mainly to analyze the types of environmental impacts and the sensitivity of inventory data, to determine the contribution of each stage to the LCA result, and to ensure the integrity of the data. The report simply draws conclusions and provides suggestions for improvement. For the interpretation step, attention should be paid to the following contents: whether sensitivity analysis was carried out on the chosen LCA method, whether uncertainty analysis was carried out on the results, and what conclusions were finally obtained.

## 4. Environmental Impact Assessment (EIA) Index System of Agricultural LCA

For the specific impact categories of environmental load, the covered categories in the EIA index system include climate change, ozone depletion, acidification, freshwater eutrophication, marine eutrophication, human toxicity, photochemical oxidant formation, particulate matter formation, terrestrial eco-toxicity, freshwater eco-toxicity, marine eco-toxicity, ionizing radiation, urban land use, and fossil energy consumption [19]. These indicators are designed and suitable for exploring the environmental impact in industrial LCA. However, the necessity of incorporating biodiversity, land use, and ecosystem services (ES) as impact categories in LCA methodologies has long been recognized by UNEP LCA initiative [20,21]. Recently, some studies have brought biodiversity, land use and ES into the agricultural LCA index system [22,23,24]. However, most of these evaluation methodologies have not been widely recognized.

### 4.1. Considering Biodiversity in Agricultural LCA Index System

Biodiversity is important to maintain or promote ecosystem functioning and stability in a changing environment [3,25,26]. However, anthropogenic activities, directly or indirectly, have caused a severe decline in biodiversity [27], among which land use is the biggest threat to biodiversity [28,29]. With the increasing demand for agricultural products and the continuous improvement of global trade level, there is an urgent need to solve the complex relationship between production and biodiversity [30], where LCA is an indispensable tool for quantitative evaluation and analysis of this complex relationship. For the past 15 years, many studies have been carried out to investigate the effect of anthropogenic activities on biodiversity decline in the framework of LCA, but there are great differences in the selection of evaluation methodologies and evaluation indicators.

In terms of evaluation methodologies, Myllyviita et al. [31] evaluated the life cycle of 1 m^3^ wood in northern and southern Finland based on the species richness index approach and ecosystem index approach. The result of the richness index approach showed that the relative biodiversity deterioration per m^3^ of wood was 0.019–0.036 in southern Finland and 0.019–0.035 in northern Finland, while the relative biodiversity deterioration was 0.041 per m^3^ of wood in Southern and 0.206 in Northern Finland when using the ecosystem indicator approach. The reason for the difference between two approaches is that the species richness approach covers all species, while the ecosystem index approach is based on endangered species. In order to quantify the effect of land use on biodiversity in LCA, Michelsen [32], for the first time, proposed the ecological quality (*Q_e_*) formula of land use based on ecosystem scarcity (n*ES*), ecosystem vulnerability (*EV*), and conditions for maintained biodiversity (*CMB*), which could be calculated as follows:

(1) $$Q_{e}=nES\times EV\times CMB$$

where, the intrinsic quality (n*ES* × *EV*) is available for all ecoregions in the world, but *CMB* factors are needed to be developed individually. However, this methodology was initially proposed to assess the impacts related to different forestry management regimes and locations and further study is needed to investigate its suitability for other ecosystems.

Jeanneret et al. [20] developed an expert scoring system for including biodiversity as a LCA impact category in agricultural production. This method could estimate the impact of agricultural management on biodiversity by using eleven indicator-species groups (flora of crops and grasslands, birds, mammals, amphibians, snails, spiders, carabids, butterflies, wild bees and grasshoppers). Different from other biodiversity assessment methods, the expert system method does not need to define the initial conditions of biodiversity. Nevertheless, its disadvantage is that the absolute value of species loss will not be calculated.

Lucas et al. [33] carried out LCA on soybean based on the potential biodiversity damage index (*BD*) in a different ecoregion (*j*) of Brazil and assessed the biodiversity damage of 804 terrestrial ecoregions in five different land use (*i*) types (managed forest, plantation, pasture, arable land, and city). The potential biodiversity damage is calculated by multiplying Characterization Factors (*CFs*) [34] and the total crop area (*A_i,j_*, m^2^) as follows:

(2) $$BD_{Regional}={\left(CF_{regional,i,j}\right)}^{*}A_{i,j}$$

Lindner et al. [35] proposed a method for assessing the impact of land use on terrestrial biodiversity based on existing studies on the impact of LCIA on land use and land use change, quantifying the impact of land use on biodiversity by applying ecological regional factors (*EF*) and an actual assessment of biodiversity status in specific sites. Land use impacts can be expressed by the following formula.

(3) $$Impact=\Delta Q_{s}\times A\times \Delta t\;$$

(4) Biodiversity=EF×BP 

(5) Biodiversity impact=EF×(1−BP) 

where, *Q_s_* is soil quality (or other influence categories), *A* is the influence area, *t* stands for time, *EF* is ecological regional factor, and *BP* is biodiversity potential.

In terms of evaluation indicators for biodiversity assessment in LCA, species richness is selected by most existing studies because of its easiness of calculation and availability of required data. In addition, some studies also use α diversity, β diversity, ecosystem scarcity, and vulnerability as evaluation indicators. For instance, Schmidt [36] calculated the species loss caused by land use based on the species-area relationship (SAR) ecological model by using α diversity as a biodiversity index in the LCIA stage. Weidema and Lindeijer [37] developed an indicator for ecosystem biodiversity (*Q_bio_*) by including species richness (n*SR*), ecosystem scarcity (n*ES*) and ecosystem vulnerability (n*EV*), which were calculated as follows:

(6) $$Q_{bio}=nSR\times nES\times nEV$$

However, a single indicator is not sufficient to quantify the effect on biodiversity due to its versatility and potential complexity. Therefore, further study is needed to improve the evaluation of biodiversity change in the EIA index system. Firstly, most studies about biodiversity decline in LCA focus on the evaluation of species richness but lack studies on species evenness and species function. Meanwhile, more research is needed to include ecological diversity, genetic diversity, population dynamics, structure and stress in the EIA index system. Secondly, relationships between species and species/habitats are oversimplified when exploring the effect on biodiversity decline, where further studies are required to better describe these complex relationships. Thirdly, different methodologies and indicators were used for different studies in considering the impact on biodiversity in agricultural LCA, which is quite controversial and difficult to compare across studies. Thus, a set of systematic and comprehensive assessment methodologies and indicators is desired.

### 4.2. Considering the Effect of Land Use and Land Use Change (LULUC) in Agricultural LCA

Agricultural land resources cover ~37.6% of the world’s land area and have been the foundation of human existence for centuries, as well as the basis for shaping cultural differences, the ecosystem balance and spatial order [38]. However, the misuse of land has resulted in a series of ecological and environmental problems, such as the degradation of production capabilities, the destruction of land structure and function, and the devastation of the environment [38,39]. Since the 1990s, several methods and tools have been developed for an integrated assessment of the sustainability of agricultural systems and land use [40] in order to address the complexity of globalized socio-political and economic contexts. For instance, SEAMLESS (a component-based framework for the European Union) includes comprehensive datasets on land use in Europe and evaluates the interaction between various agro-technologies and land use [41]. The SENSOR project developed ex-ante Sustainability Impact Assessment Tools (SIA Tool) to estimate the benefits and trade-offs of land use changes [42]. The PLUREL (Peri-urban Land Use Relationships) project developed tools to identify strategies for sustainable urban development and urban-rural linkages of land use [43], while the MATISSE project developed a model for assessing transitions to sustainable mobility [44]. Furthermore, with the rapid development of the Harmonized World Soil Database (HWSD) and GIS spatial analysis technology, the evaluation of land use impacts has become a priority category in LCA in recent years [45].

To date, most studies considering the effect of LULUC in agricultural LCA mainly assess its impacts on life support functions (LSF) [46] or biodiversity [47]. The LSF concerns the role that an ecosystem plays in maintaining life processes, where free net primary production (fNPP) [48] or soil organic matter (SOM) [46] could be used as an indicator. Although SOM cannot fully consider all aspects of soil functioning, it is a robust indicator for soil quality for its flexibility in data collection and has been considered as a common indicator of land use impact assessment in LCA [49]. To evaluate the impact of LULUC on biodiversity, species diversity [48] or species richness [50] were often chosen as an indicator. Chaudhary et al. [51] and the UNEP/SETAC Life Cycle Initiative [52] further proposed an approach to assess biodiversity impacts by using the countryside species–area relationship (SAR) model to predict loss of species for each of the five taxonomic groups (mammals, birds, vascular plants, amphibians and reptiles) in 804 terrestrial ecoregions and provided the aggregated characterization factors (CFs) for biodiversity. To evaluate the impact of LULUC on ecosystem services in LCA, Othoniel et al. [53] proposed a new methodology to calculate the midpoint and end-point CFs, where the calculated CFs enable us to assess the effect of six land cover types on six ecosystem functions and two final ecosystem services, and to identify spatial trade-offs and synergies between ES due to LULUC. Chaplin-Kramer et al. [54] integrated spatially explicit modelling of land change and ES in a Land-Use Change Improved (LUCI)-LCA and found that the LUCI-LCA approach resulted in opposite results compared to standard LCA for GHG emissions and water consumption. The LUCI-LCA approach firstly used the land-change model based on logistic regression with climatic and soil suitability to predict plausible future agricultural LULUC, rather than a prediction based on current status in standard LCA. Moreover, the LUCI-LCA approach translated LULUC impacts by using spatially explicit models for biodiversity (GLOBIO) [55] and ES (InVEST) [56], rather than linear relationships between impacts and production in standard LCA.

### 4.3. Considering the Ecosystem Service (ES) in Agricultural LCA

Ecosystem services (ES) are defined as the directly or indirectly obtained benefits by humankind (human welfare) from ecosystem functions and consist of material, energy and information flows from natural capital [57,58], which are normally divided into four categories: namely, provisioning services, regulating services, cultural services, and supporting services [59]. There are three commonly used classification systems to assess ES worldwide, including Millennium ecosystem Assessment (MA) [59], the Economics of Ecosystems and Biodiversity (TEEB) [60], and Common International Classification of Ecosystem Services (CICES) [61].

Recently, ES has gained more and more attention in environmental impact assessment, especially in LCA [54,58,62,63,64,65,66,67,68]. Based on the CICES framework, Maia de Souza et al. [22] proposed a conceptual framework to link the Ecosystem Service Cascade Model and the LCA approach, where five level schemes (structure, function, services, benefits and values) were included. This approach enables us to identify the ES flow between human beings and ecological or biophysical processes, which provide a better view of the spatial variability and scales that ES are provided. Liu and Bakshi [64] proposed the techno-ecological synergy in LCA (TES-LCA) approach, which enables the calculation of absolute environmental sustainability metrics by considering the ES of carbon sequestration, air quality regulation and water provisioning. The TES-LCA approach included the role of ecosystem goods and services and the interdependence between technological and ecological systems in each step of LCA at multiple spatial scales, which informs us how to implement efforts at local and service shed scales for each process in LCA. Pavan and Ometto [58] proposed a new conceptual framework for soil ES assessment in LCA by including the main soil processes (biological, physical, geochemical, supporting and degradation), functions (biomass growth, raw materials source, nutrient dynamics and substances cycling, carbon balance, biological activity and productivity support, groundwater production, water regulation, platform for human activities, and landscape and culture historical continuity), services (CICES classes), benefits (harvested yield, employment, maintenance, flood and flow control carbon stocks and sequestration, avoided erosion, energy production, recreation, cultural continuity, source of knowledge), and values (market values, intrinsic values, avoided costs, social value, cultural heritage values, value of science and education). Boone et al. [65] proposed an allocation procedure to divide the environmental impact over different outputs, including provisioning and other ES, based on the capacity of agricultural systems to deliver ES, where the allocation factors, including supply service allocation factor and regulation service allocation factor, were calculated according Burkhard et al. [69]. Therefore, by considering ES in agricultural LCA, a more comprehensive understating could be achieved by assessing the environmental costs and benefits related to human activities. Alejandre et al. [66] proposed an optimal coverage of ES in LCA consisting of 15 categories based on the CICES V5.1 classification method, among which, however, only four categories of “regulation of flows and protection from extreme events”, “mediations of wastes, toxics and nuisances”, “water conditions” and “aesthetic value” were considered in LCA studies. By using bibliometric mapping and network analysis, VanderWilde and Newell [70] comprehensively reviewed decades of research on ES and LCA and also found that LCA studies focused more on a relatively small number of regulation and maintenance ES (e.g., carbon balance, carbon sequestration and carbon emissions) but less on provisioning ES and scarce on cultural services. Therefore, further research is required to develop an operational methodology and indicators to integrate missing categories of ES in LCA.

## 5. Current Progress of LCA in Different Agricultural Systems

### 5.1. Application of LCA in Intensive Agriculture

Since the 1960s, intensive agriculture has emerged to meet the growing demands for food, feed and fuel, which was characterized by land management pursuing high productivity through the use of fertilizers, pesticides, irrigation and mechanization [71]. With the rapid development of intensive agriculture, however, it also led to increased resource consumption, erosion, and widespread pollution [65]. To clarify the resource depletion issues and environmental impacts induced by intensive agriculture, LCA has been widely applied in intensive agriculture systems [9,72,73]. For instance, Pelletier-Guittier et al. [74] evaluated the attributes of hedgerows used by mammals under an intensive agricultural landscape and showed that hedgerows were beneficial for wildlife conservation in intensive agricultural landscapes. To assess the environmental impact of different wheat production systems with different fertilization intensities and types, Charles et al. [75] conducted a LCA by considering both yield and quality of the product and found that optimal combinations of variety, fertilization and land use are necessary for the best production strategies design. Lares-Orozco et al. [76] used LCA to quantify the GHG emissions at different stages of wheat production in the intensive agricultural ecosystem of the Yaqui Valley in Mexico, and found that fertilization was the main source of GHG in wheat production, accounting for 83% of the life cycle emissions, and that GHG emissions from conventional tillage and no tillage can be reduced by 33% and 24%, respectively, by using more efficient tractors with decreased diesel inputs. Wu et al. [77] evaluated the GHG emissions among nine crops with seven different chemical fertilizer production and use processes from 1998 to 2016 in China by a partial LCA approach and found that the total GHG emissions increased by 35% over 18 years, with more than half of it from chemical fertilizer use. The three main grain crops: rice, wheat, and maize, contributed half of the total GHG emissions due to their larger cultivated areas, while urea generated the greatest GHG emissions among different chemical fertilizers. Therefore, to mitigate the GHG emissions, different combinations of chemical fertilizer and crops should be taken into consideration.

### 5.2. Application of LCA in Organic Agriculture

Organic agriculture has been proposed as a solution to reduce the negative impact of intensive agriculture on the environment since the 1940s, when Albert Howard adopted Lord Northbourne’s terminology of “organic farming” and published *The Soil and Health: A Study of Organic Agriculture* [78]. Organic agriculture is defined as a production system that sustains the health of soils, ecosystems, and people, relying on benefits from ecological processes, biodiversity and natural pest control rather than synthetically produced inputs (fertilizers and pesticides). Organic agriculture often results in a reduction in GHG emissions and the promotion of biodiversity, water use efficiency, and soil, water, and air quality [65,79]. However, it can also lead to a yield reduction of 20–40% for arable crops when compared with conventional systems [80,81,82]. Therefore, LCA, as a comprehensive environmental assessment tool, has been widely applied in comparison to organic farming and conventional farming systems.

Tricase et al. [83] evaluated barley yield and the environmental sustainability of conventional and organic agriculture by using LCA and found that organic barley cultivation is the most environmentally sustainable solution but not efficient in production. Cederberg and Mattsson [84] compared the environmental impact of milk production under conventional and organic farming in Sweden by using the LCA method, including three categories of environmental impact, i.e., resources (energy, materials, and land use), human health (pesticide use), and ecological effects (global warming, acidification, eutrophication, ozone formation). Their results showed that organic farming has obvious environmental benefits such as the reduced use of pesticides and phosphorus. Meisterling et al. [85] found that the global warming potential (GWP) of organic wheat (30 g CO_2_-eq per kg) was less than that of conventional wheat by conducting a LCA including agricultural inputs, grain farming and transport processes. Knudsen et al. [86] compared the carbon footprints of different organic agricultural systems with different sources of N supply (slurry, biogas and mulching) and found that the biogas rotation, assuming biogas replaces fossil gas, showed a significantly lower carbon footprint per kg crop than other systems. Meier et al. [87] reviewed 34 comparative LCA studies of organic and conventional agricultural products and found that most studies only focused on the harvested products and only a limited number of impact categories are assessed within the impact assessment, which does not allow for a comprehensive environmental assessment. Agriculture not only provides products, but also delivers multiple ecosystem services besides provisioning services [65,87]. However, some key aspects of sustainable agriculture, such as better soil health and biodiversity, are largely ignored in current LCA methodology [88]. Therefore, more research is needed to include more operational indicators and a broader perspective of agricultural multi-functionality towards a better representation of organic agriculture in LCA.

## 6. Integrated Application of LCA and Other Models

The LCA method is limited to the assessment of the environmental impacts, but pays less attention to the social, economic, and other aspects of agricultural systems. Therefore, combining a variety of evaluation methods to comprehensively assess different aspects of the agroecosystem is highly required, which can not only expand the scope of assessment, but also complement different models to increase the effectiveness of assessment results.

### 6.1. Integration of Agricultural LCA and Agent Based Modeling (ABM)

LCA has been widely used to assess the environmental impacts of agricultural activities, but it lacks attention to the temporal and spatial dynamics of agricultural systems given that the traditional LCA method treats a system as static [89]. For instance, crop selection and fertilizer application in rotation system, pollutant discharge time and release rate can vary largely in different periods and regions. To overcome these shortcomings, the integration of LCA and ABM could provide a potential assessment of environmental impacts in dynamic agricultural systems. ABM is a computational model specially designed to simulate the actions and interactions of autonomous agents (teams, organizations) [90], which has been widely used in modeling agricultural systems of climate adaptation and mitigation options [91], land use change [92], and so on. The integration of LCA–ABM models could include human behavior and local variabilities in the studied system [93], and a few studies have used LCA–ABM to evaluate sustainability in agriculture systems [94,95,96]. Marvuglia et al. [94] used ABM to simulate future crop patterns in Luxembourg under a pre-defined scenario, which introduced a “green consciousness” component in farmers’ decision and performed a LCA of its agricultural system by using ABM results. Gutiérrez et al. [95] explored the influence of farmers’ environmental awareness on the environmental impacts linked to farming activities by using LCA to calculate the CO_2_ emissions, a criterion in farmers’ decision making an ABM. Bichraoui-Draper et al. [96] applied an ABM-LCA approach to model farmers’ potential adoption of switchgrass as a biomass and calculate their CO2 emissions of each farm based on their decisions. The results showed that economic situation and crop prices are the most influential factors for farmers’ decision. Lan and Yao [89] integrated LCA, ABM, and Techno-Economic Analysis (TEA) and evaluated the environmental impacts of an agricultural system including 1000 farms over a 30-year time period under changing climate and economic conditions. Their results suggested that access to environmental information, information exchange among farmers, farmers’ environmental awareness, and farm size were key factors driving the system’s environmental impacts, which showed the potential to assess sustainability in dynamic systems, involving human behaviors.

Although an ABM compensates for spatial-temporal dynamics in the LCA model, there are still some aspects that need to be improved. The integration of LCA and ABM may increase the probability of parameter uncertainty, but little research has been carried out to develop feasible mathematical methods to quantify this uncertainty. Due to the nature of the ABM (non-linear computational model) and LCA (linear deterministic method), the coupled ABM-LCA approach adds new uncertainty sources to the LCI and limits the applicability of propagation methods, since it cannot be expressed as an explicit formula [93]. Therefore, an operational framework is highly required, which can flexibly deal with different coupling options of two models and propose adequate methods for different uncertainty sources, including uncertainty of model structure and uncertainty due to choices.

### 6.2. Integration of Agricultural LCA and Data Envelopment Analysis (DEA)

Although LCA has been widely used as a standard methodology for environmental impact analysis, it encounters problems when several units are needed for the inventory [97]. Data Envelopment Analysis (DEA), a linear programming model expressed as the ratio of output to input, is widely used in the evaluation of the productive efficiency of multiple similar entities [98]. Therefore, the integration of LCA and DEA was highly encouraged when data are available for multiple similar entities [99,100,101]. 

Masuda [102] evaluated the ecological efficiency of intensive rice production in Japan by combining LCA and DEA methods, where GWP and eutrophication from the LCA results were used as inputs for DEA and weight-based rice yield was output from DEA. The results showed that expanding the size of rice farms is an effective way of improving the ecological efficiency of intensive rice production in Japan. Pishgar-Komleh et al. [103] combined DEA and LCA to evaluate the environmental efficiency of winter wheat cropping system in Poland by selecting an appropriate DEA model. The results showed that a slack based measure (SBM)-DEA with undesirable outputs could better reflect the performance of undesirable outputs and that the evaluated efficiency of winter wheat farm was 0.43 and improving the low efficiency farm to the high efficiency farm could save 57% of resources. Zhong et al. [104] evaluated the ecological economic efficiency of oasis agriculture in the arid areas of northwest China by combining the LCA and DEA model, where maize seed farmers were selected as the research object, and LCA is used to quantitatively analyze the environmental impacts in the seed maize production. The normalized results of LCA were then used as input for SBM-DEA model. The results showed that the average ecological economic efficiency is 0.871 in the studied oasis agriculture.

The integration of LCA and DEA comprehensively assess the environmental and operational performance of multiple similar entities, achieving the verification of ecological efficiency, as well as avoiding using the average inventory data (i.e., to avoid the standard deviation), which could eliminate many subjective factors. A major limitation of the conventional LCA method is that it can only evaluate environmental effects, while the joint application of LCA and DEA can simultaneously evaluate the environmental and economic benefits of the agricultural production process, which is an important tool to achieve sustainable development goals. Therefore, further studies on the integration of LCA and DEA is expected for the assessment of multiple units’ performance due to the numerous strengths of this methodology [101].

### 6.3. Complementary Applications of LCA with Other Models

As an effective environmental management tool, LCA is characterized as a standard approach accounting for resource consumption and environmental impacts of agricultural production at different stages of the whole life cycle. To make it more operational and reliable, more and more studies have combined different models with LCA to increase the effectiveness of the assessment results.

Emergy analysis (EMA), which integrates resource inputs, energy inputs, and changes in energy stored in the system into solar energy [105,106], has been widely used to quantitatively evaluate system resource utilization efficiency, environmental load and potential sustainability [107,108]. EMA includes energy flow, material flow and money flows in the system, which compensates for the shortcomings of LCA that does not consider economic and human resources aspects. Furthermore, EMA includes all direct and indirect inputs, especially freely available renewable resources (such as sunlight, rain, wind, and earth circle), which are typically ignored in LCA. Therefore, the synergistic application of EMA and LCA offered a comprehensive donor-side perspective [109], which is far more effective than the single application of either method [110,111].

The weighting of different impact categories is a commonly used approach in LCA, but ISO standard of LCA [6] does not recommend weighting in comparative studies. Therefore, LCA is sometimes criticized for making subjective choices. Participatory methods and/or Multi-Criteria Decision Analysis (MCDA), a family of methods that help decision makers identify and select a preferred alternative when faced with a complex decision-making problem characterized by multiple objectives [112], has been used for weighting in LCA to integrate and balance different sustainability dimensions [113,114,115]. Jouini et al. [116] constructed an operational framework integrating LCA and participatory approach in the evaluation of environmental impacts in rural areas of developing countries with limited available data. This integrated framework uses inventory data and local knowledge provided by stakeholders to perform LCA, which further involves all the stakeholders and considers all interests, values, and the diversity of social representations in the territory. The joint application of LCA and MCDA methods could provide additional insights for LCA evaluation, such as the selection of sustainability indicators and the weighting of indicator-specific results [117,118].

## 7. Prospect and Challenges of Agricultural LCA

The agricultural sector, providing a large number of products and services, is vital to human beings. With the increasing requirement to feed 7.9 billion people, the agroecosystem comes with a huge environmental cost [3,4]. Agricultural LCA has been seen as an effective way to assess resource consumption and environmental burdens in the whole process of agricultural production or agricultural activities. However, despite the increasing literature concerning agricultural LCA, both on its methodological aspects and in case studies, several prospects and challenges still need to be addressed in agricultural LCA to improve its robustness.

Firstly, the construction of a regional heterogeneity database and innovated methodologies are needed. Due to the global, regional, and even local variations in management practices, it is difficult to conduct agricultural LCA based on general globally database. Furthermore, collecting a large number of regionally heterogeneity datasets may lead to high cost. Therefore, how to represent relevant variability in LCA study without collecting a huge range of data requires innovated methodologies based on limited data requirement to reflect regional variations in agricultural LCA. Furthermore, the necessity of incorporating biodiversity, land use, and ecosystem services (ES) as impact categories in LCA methodologies has long been recognized to improve the accuracy of the regional specificity evaluation of agricultural LCA.

Secondly, there is a clear need for more meaningful FUs for agricultural products, for instance, covering the nutritional function of food [7,8], social dimensions of products (e.g., wine, beers or coffee) [9,10], or even the cultural function of agriculture (i.e., custodian of cultural and natural heritage) [119]. Therefore, there is a need to address environmental impacts that are not well addressed in current LCA studies by proposing a more sophisticated way of defining FU, which may improve and enlarge LCA and integrate knowledge from other domains.

Furthermore, agricultural LCA should be complemented by other models to increase the effectiveness of assessment results. The structure of the agricultural system is largely influenced by consumers’ choice, but some aspects, including economics and societal concerns, are out of the LCA framework. Therefore, the integrated application of LCA and other models may make it possible to consider different aspects that influence the choice of a product and further expand the scope of LCA evaluation. Future research on these prospects and challenges will make agricultural LCA more robust and effective in environmental impacts assessment to support decision making, from individual farms to regional or (inter)national, for the sustainable future of agriculture.
